# The Landscape of Bispecific Antibodies in Solid Tumor Oncology: Trends, Challenges, and Opportunities

**DOI:** 10.1002/cam4.71432

**Published:** 2025-12-12

**Authors:** Rafik ElBeblawy, Chinmay Jani, Judith Pérez‐Granado, Mark Gramling, Aakash Desai

**Affiliations:** ^1^ Huntsville Hospital Health System, Decatur Morgan Hospital Decatur Alabama USA; ^2^ University of Miami Sylvester Comprehensive Cancer Center Miami Florida USA; ^3^ The Larvol Group, LLC San Francisco California USA; ^4^ The O'Neal Comprehensive Cancer Center at the University of Alabama at Birmingham Birmingham Alabama USA

**Keywords:** antibody‐drug conjugates, biomarkers, bispecific antibodies, clinical trials, immune checkpoints, solid tumor oncology, therapeutic landscape

## Abstract

**Background:**

Bispecific Antibodies (BsAbs) represent a novel class of immunoglobulins that can target and bind to two distinct antigens simultaneously. The global BsAbs market, valued at approximately USD 8.65 billion in 2023, reflects tremendous promise and substantial growth. Here, we present a comprehensive analysis of the current BsAbs research pipeline in solid tumors.

**Methods:**

Data were extracted from LARVOL CLIN, an outcomes database storing over 130,000 trials, to analyze the number of BsAbs including the number of trials per company, the timeline of trial initiation, phases, specific molecules, targets, and associated biomarkers. LARVOL CLIN leverages regular expression‐based text mining to extract data from major trial registries, including ClinicalTrials.gov, EudraCT, the Australian registry ANZCTR and the Asian Registries ChiCTR and UMIN.

**Results:**

Our analysis identified 681 registered clinical trials in ClinicalTrials.gov, a number that has doubled since 2019, each with a known target and phase, evaluating a total of 183 BsAbs. Solid tumors, particularly gastrointestinal, lung, gynecological and breast cancer, have emerged as primary focuses. A significant trend is the strong focus on immune checkpoint bispecific targets, particularly PD1‐CTLA4 (*n* = 216 trials, 31.7%, 4 BsAbs), PD1‐VEGF (*n* = 56 trials, 8.2%, 3 BsAbs), followed by oncogene targets like EGFR‐MET (*n* = 34 trials, 5%, 3 BsAbs). The distribution of clinical molecules also reflects such focus, with dual PD1‐CTLA4 inhibitors (Cadonilimab, Volrustomig, Danviostomig) leading the highest number of trials (*n* = 208, 30.5%). Other promising molecules include Ivonescimab (*n* = 51, 7.5%), targeting PD1‐VEGF, and Amivantamab‐vmjw (*n* = 29, 4.3%), targeting EGFR‐MET. Clinical trials investigating PD1‐CTLA4 BsAbs showed improved overall survival (OS) and progression‐free survival (PFS) with tolerable adverse effects when combined with standard chemotherapy. Only 38% of trials, *n* = 258, specified a biomarker inclusion. Among oncogene‐targeted BsAbs, *EGFR* is the most prevalent (*n* = 80, 31%), followed by *HER2* (*n* = 77, 29.8%), and *ALK* (*n* = 41, 15.8%). Gene‐specific alterations were also represented; *HER2*‐positive alterations garnered the most attention in *n* = 35 trials. BsAbs development is primarily driven by biotechnology companies (Akesobio, Alphamab, Biokin Pharma, etc.) compared to large pharmaceutical companies (J&J, AstraZeneca, and Boehringer Ingelheim). Geographically, the United States, China and the European Union have the highest number of trials available.

**Conclusion:**

Since the first BsAb approval in 2014, the field has rapidly expanded, with solid tumor oncology advancing dynamically. The major focus has been on combining BsAbs with immunotherapy strategies, followed by targeting known oncogenic pathways. The shift toward biotechnology‐led innovation underscores the growing therapeutic and financial interest in this field. Optimizing the efficacy and safety of these molecules is key to paving the way for the next era of immune and precision oncology.

## 1 Background

The approval of immune checkpoint inhibitors (ICIs) has revolutionized oncology care, establishing immunotherapy as a cornerstone in cancer treatment [1]. These therapies, by disrupting inhibitory signaling pathways such as PD‐1/PD‐L1 and CTLA‐4, have demonstrated unprecedented efficacy in a subset of patients across multiple cancer types [2]. However, the benefits are not universal, with many patients exhibiting intrinsic or acquired resistance.

Bispecific Antibodies (BsAbs) have emerged as a compelling class of immunotherapeutic agents that offer novel mechanisms of action and can be utilized in different sequencing or combination strategies to overcome this resistance. They simultaneously bind to two different antigens, often a tumor‐associated antigen and an immune effector molecule such as CD3. BsAbs enable direct immune cell engagement and targeted tumor cell elimination [3]. This dual‐targeting capability enhances tumor selectivity, reduces the likelihood of immune escape, and facilitates the formation of immunologic synapses that can potentiate T‐cell cytotoxicity within the tumor microenvironment (TME) [4]. BsAbs are especially valuable in poorly immunogenic “cold tumors” where ICIs may have limited impact due to poor immune infiltration [4].

Since the first BsAbs approval in 2014, the field has undergone rapid expansion, particularly within the domain of treatment for solid tumors [5, 6]. In 2023, the BsAbs market was valued at approximately USD 8.65 billion, underscoring both the growing clinical interest and investment enthusiasm surrounding this therapeutic class [7]. Advances in bioengineering including improved stability, Fc‐fusion strategies, and modular design, have supported the development of diverse BsAbs formats [8]. These innovations have translated into a surge of clinical activity, with an increasing number of BsAbs entering both early and late‐phase trials aimed at advanced solid malignancies [5].

In this article, we provide a comprehensive analysis of the current BsAbs research pipeline in the treatment of solid tumors. We explore trends in trial design and volume, molecular target selection, and the competitive landscape, including leading companies and the geographical distribution of innovation. This review highlights key scientific and clinical opportunities while outlining the challenges that must be addressed to fully realize the potential of BsAbs in solid tumor immunotherapy.

## 2 Methods

We utilized data extracted from LARVOL CLIN (https://clin.larvol.com), a clinical outcomes database that aggregates information from major clinical trial registries, including ClinicalTrials.gov, EudraCT, ANZCTR, ChiCTR, and UMIN—totaling coverage of 130,000 trials. Using regular expression–based text mining, Artificial Intelligence and manual curation, the database systematically captures detailed information and publicly available outcomes data across all tumor types and indications. Specifically, it applies natural language processing (NLP) methods including large language models (LLMs) and named entity recognition (NER) to extract key trial attributes such as disease, settings, staging, biomarkers, endpoints, and hazard ratios.

Our analysis examined key aspects of the BsAbs landscape in solid tumors, including trial volume, initiation timelines, and longitudinal trends. We further assessed sponsor distribution, development phases, and pipeline maturity across industry players while identifying major antigen targets and biomarkers.

## 3 Results

### 3.1 Trial Distribution

**FIGURE 1:**
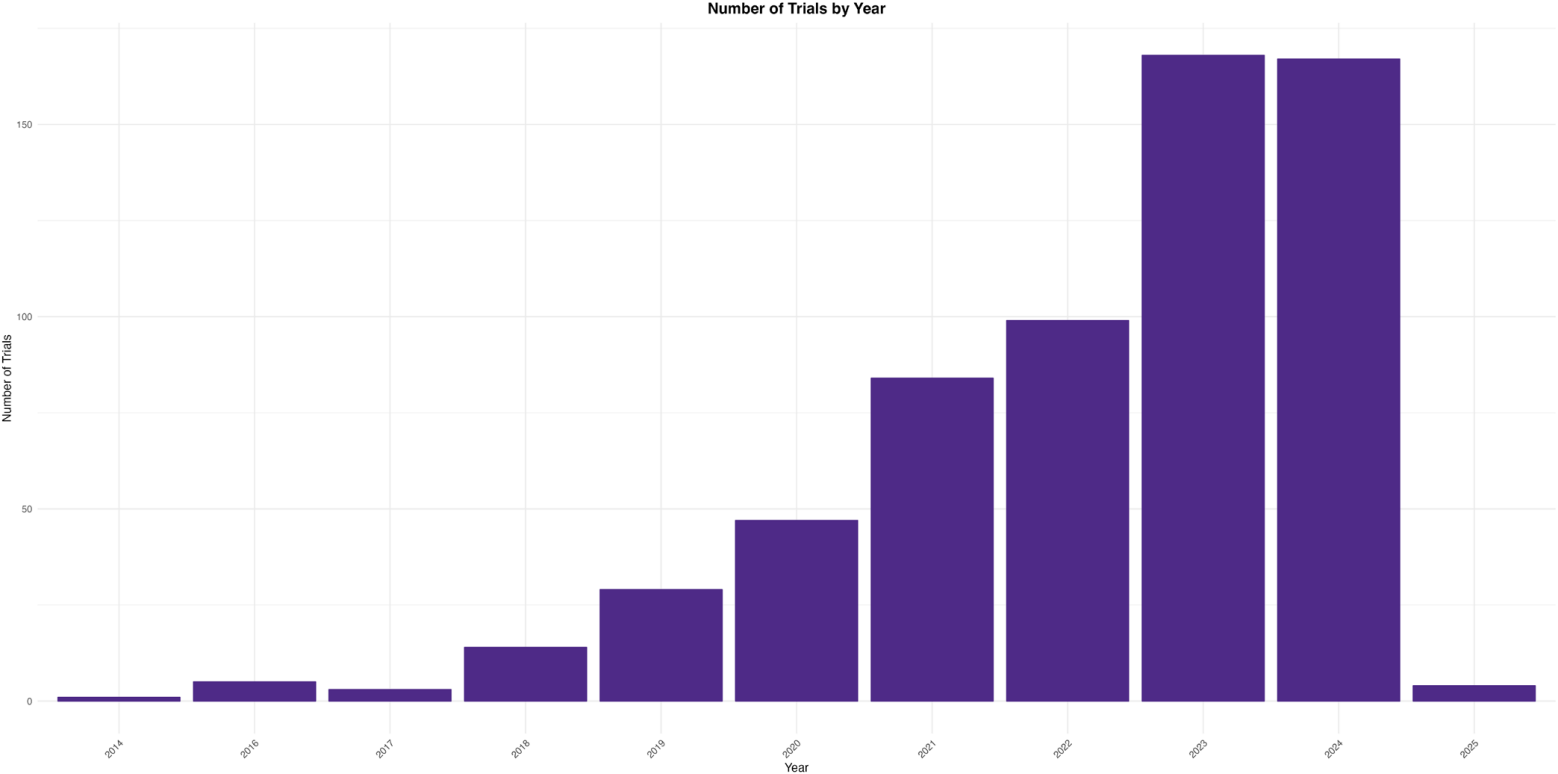
Trend of number of BsAbs clinical trials stratified by year of the study.

We identified 681 registered clinical trials on ClinicalTrials.gov evaluating a total of 183 BsAbs, each with a known molecular target and study phase. Notably, this figure has doubled since 2019, highlighting the rapid acceleration in BsAbs clinical development. Between 2014 and 2025, there was a steady increase in the number of registered trials. A single trial was documented in 2014, followed by five trials in 2016. A gradual upward trend was observed thereafter. In 2021, a marked surge with 84 trials represented a sevenfold increase compared to 2018. By 2024, the number of trials had risen to 167, underscoring the sustained and growing momentum in BsAbs research (Figure 1; Table 1).

**TABLE 1:** Number of trials per year.

Start date	Trial count
2014	1
2016	5
2017	3
2018	14
2019	29
2020	47
2021	84
2022	99
2023	168
2024	167
2025	4

**FIGURE 2:**
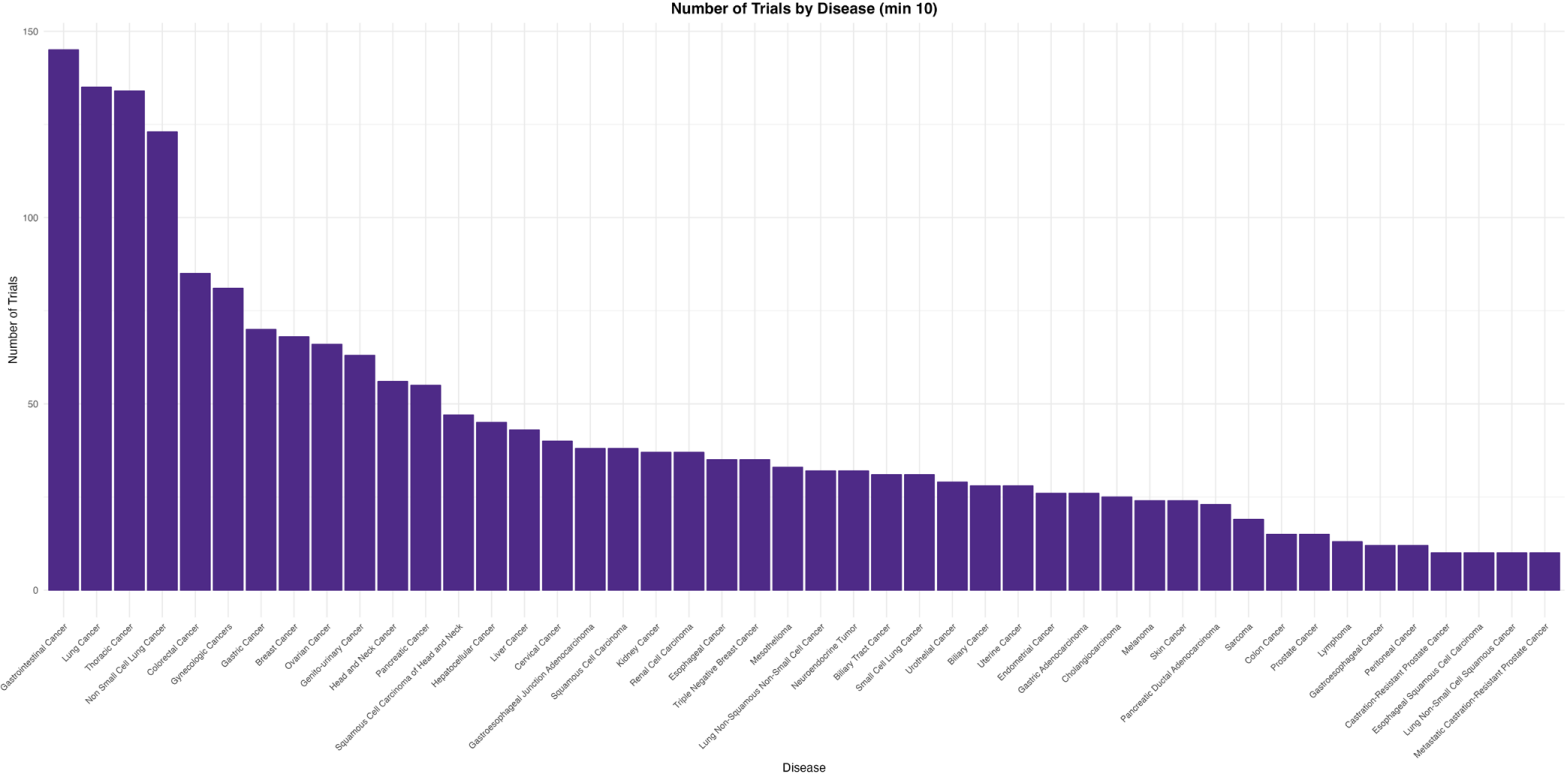
Number of trials stratified by type of malignancy.

Among specific cancer types, Gastrointestinal cancers had the greatest representation *n* = 145 trials (21.3%), followed by Lung Cancer *n* = 135 (19.8%) and Colorectal Cancer *n* = 85 (12.5%). Substantial trial development efforts were also observed in relatively less common cancers such as Neuroendocrine Tumors *n* = 32 (0.05%) and Mesothelioma *n* = 33 (0.05%). While fewer in number, trials for hematologic malignancies, including Lymphoma *n* = 13, Diffuse Large B‐Cell Lymphoma *n* = 7, and Hodgkin Lymphoma *n* = 5, were also present (Figure 2).

**FIGURE 3:**
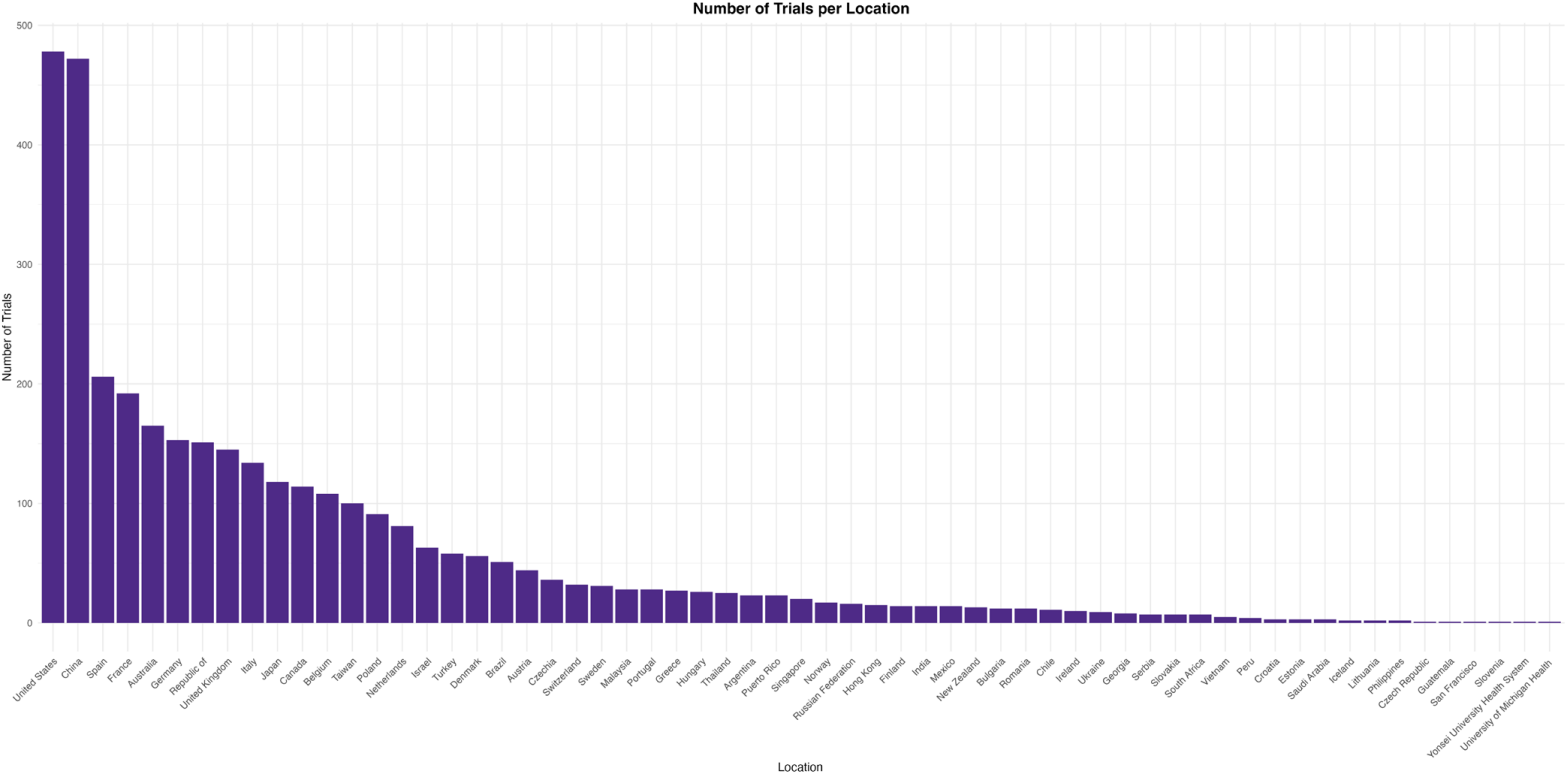
Number of trials per country worldwide.

Geographically, the United States and China, with 478 and 472 trials respectively, dominated the contribution to the emerging BsAbs research. The European Union followed, led by Spain (*n* = 206), France (*n* = 192), and Germany (*n* = 153) trials. Low and middle‐income countries like Brazil, Malaysia, and India each conducted fewer than 50 trials (Figure 3; Table 2).

**TABLE 2:** Number of trials per country.

Locations	Trial count
United States	478
China	472
Spain	206
France	192
Australia	165
Germany	153
Republic of	151
United Kingdom	145
Italy	134
Japan	118
Canada	114
Belgium	108
Taiwan	100
Poland	91
Netherlands	81
Israel	63
Turkey	58
Denmark	56
Brazil	51
Austria	44
Czechia	36
Switzerland	32
Sweden	31
Malaysia	28
Portugal	28
Greece	27
Hungary	26
Thailand	25
Argentina	23
Puerto Rico	23
Singapore	20
Norway	17
Russian Federation	16
Hong Kong	15
Finland	14
India	14
Mexico	14
New Zealand	13
Bulgaria	12
Romania	12
Chile	11
Ireland	10
Ukraine	9
Georgia	8
Serbia	7
Slovakia	7
South Africa	7
Vietnam	5
Peru	4
Croatia	3
Estonia	3
Saudi Arabia	3
Iceland	2
Lithuania	2
Philippines	2
Czech Republic	1
Guatemala	1
San Francisco	1
Slovenia	1
Yonsei University Health System	1
University of Michigan Health	1

### 3.2 Targets and Biomarkers

**FIGURE 4:**
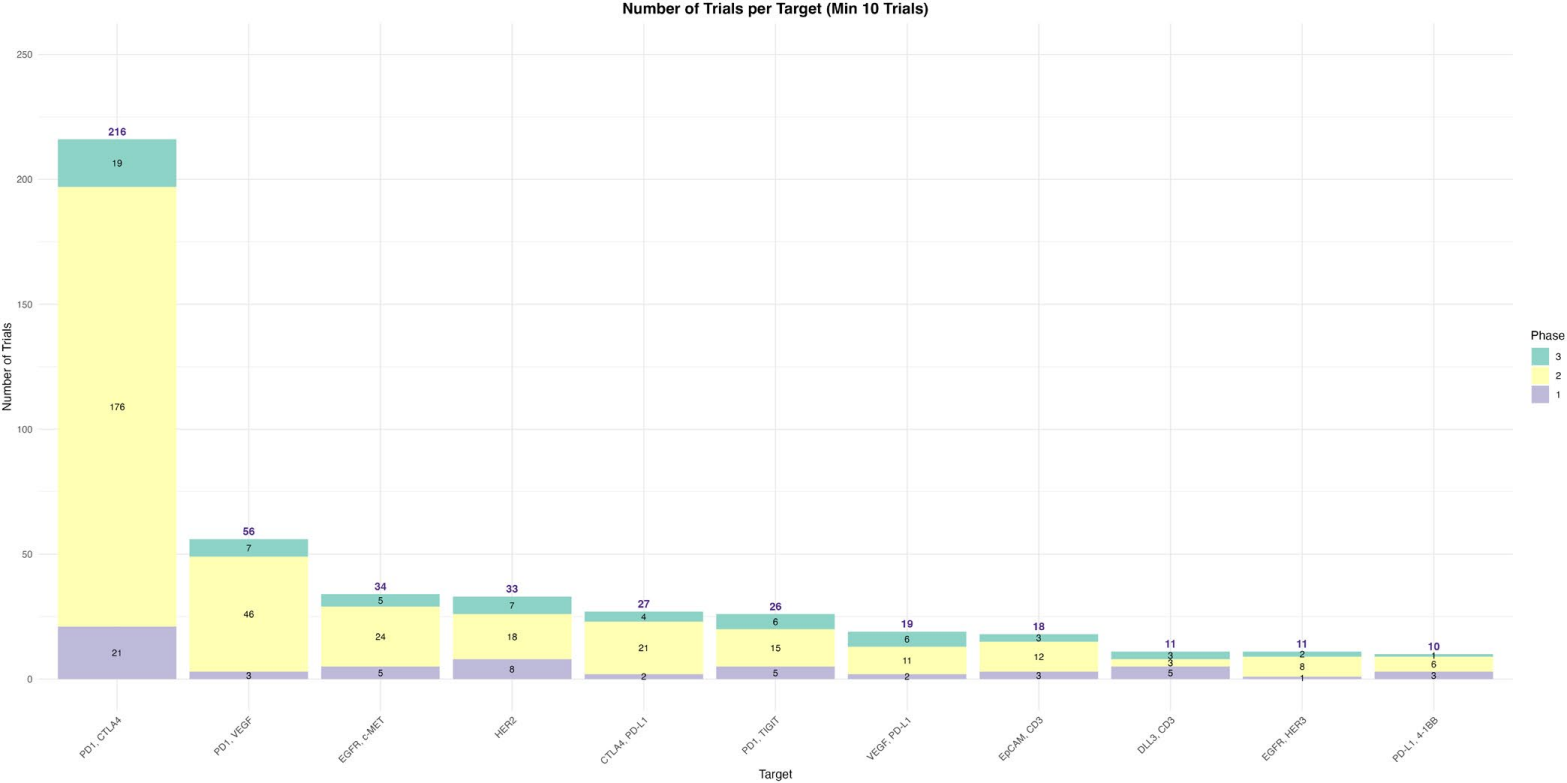
Number of trials stratified by combination target.

We found that the BsAbs development is heavily concentrated around a few dominant target combinations, particularly immune checkpoints. PD‐1/CTLA‐4 represented the most common target with *n* = 216 trials (31.7%), and 4 BsAbs. PD‐1/VEGF follows as the second most pursued target with *n* = 56 trials (8.2%) and 3 BsAbs. Other notable targets include EGFR/c‐MET (*n* = 34 trials, 5.0%), HER2 (*n* = 33 trials, 4.8%) and CTLA‐4/PD‐L1 (*n* = 27 trials, 4.0%). Additional targets such as PD‐1/TIGIT (*n* = 26 trials, 3.8%) and VEGF/PD‐L1 (*n* = 19 trials, 2.8%) suggest diversification into newer immunologic axes. Although less frequent, targets like EpCAM/CD3, DLL3/CD3 and EGFR/HER3, each with fewer than 20 trials, still demonstrate the expanding scope of BsAbs innovation. Overall, only 11 targets from a total of 132 unique target combinations were represented in 10 or more trials, while the rest of the targets made less than 1.4% contribution in the current research market (Figure 4; Table 3).

**TABLE 3:** Number of trials per target and phases.

Target	Phase	Count of trials	Total number of trials
PD1, CTLA4	1	21	216
PD1, CTLA4	2	176	216
PD1, CTLA4	3	19	216
PD1, VEGF	1	3	56
PD1, VEGF	2	46	56
PD1, VEGF	3	7	56
EGFR, c‐MET	1	5	34
EGFR, c‐MET	2	24	34
EGFR, c‐MET	3	5	34
HER2	1	8	33
HER2	2	18	33
HER2	3	7	33
CTLA4, PD‐L1	1	2	27
CTLA4, PD‐L1	2	21	27
CTLA4, PD‐L1	3	4	27
PD1, TIGIT	1	5	26
PD1, TIGIT	2	15	26
PD1, TIGIT	3	6	26
VEGF, PD‐L1	1	2	19
VEGF, PD‐L1	2	11	19
VEGF, PD‐L1	3	6	19
EpCAM, CD3	1	3	18
EpCAM, CD3	2	12	18
EpCAM, CD3	3	3	18
DLL3, CD3	1	5	11
DLL3, CD3	2	3	11
DLL3, CD3	3	3	11
EGFR, HER3	1	1	11
EGFR, HER3	2	8	11
EGFR, HER3	3	2	11
PD‐L1, 4‐1BB	1	3	10
PD‐L1, 4‐1BB	2	6	10
PD‐L1, 4‐1BB	3	1	10
CD3, DLL3	1	7	9
CD3, DLL3	2	2	9
c‐MET, EGFR	1	5	9
c‐MET, EGFR	2	3	9
c‐MET, EGFR	3	1	9
PD1, LAG‐3	1	2	8
PD1, LAG‐3	2	4	8
PD1, LAG‐3	3	2	8
ANG2, VEGF	1	5	7
ANG2, VEGF	2	2	7
PD‐L1, PD1	1	6	7
PD‐L1, PD1	2	1	7
PD1, TIM‐3	1	3	6
PD1, TIM‐3	2	3	6
VEGF, DLL4	2	5	6
VEGF, DLL4	3	1	6
VEGF, PD1	1	2	6
VEGF, PD1	2	4	6
CD3, EpCAM	1	2	5
CD3, EpCAM	2	2	5
CD3, EpCAM	3	1	5
CTLA4, PD1	1	2	5
CTLA4, PD1	2	3	5
DLL4, VEGF	1	3	5
DLL4, VEGF	2	1	5
DLL4, VEGF	3	1	5
TIGIT, PVRIG	1	4	5
TIGIT, PVRIG	2	1	5
4‐1BB, PD‐L1	1	1	4
4‐1BB, PD‐L1	2	3	4
CD28, PSMA	1	1	4
CD28, PSMA	2	3	4
CD3, CLDN18.2	1	2	4
CD3, CLDN18.2	2	2	4
CD3, MUC16	2	4	4
CD3, PSMA	1	3	4
CD3, PSMA	2	1	4
CD3, STEAP1	1	3	4
CD3, STEAP1	3	1	4
CD47, PD‐L1	1	3	4
CD47, PD‐L1	2	1	4
HER2, PD1	1	3	4
HER2, PD1	2	1	4
PD‐L1, CD47	2	4	4
PD‐L1, LAG‐3	1	2	4
PD‐L1, LAG‐3	2	2	4
PD1, CD47	1	2	4
PD1, CD47	2	2	4
4‐1BB, EGFR	1	3	3
CD3, GPC3	1	2	3
CD3, GPC3	2	1	3
CD39, TGF‐ß	1	2	3
CD39, TGF‐ß	2	1	3
CD40, 4‐1BB	1	1	3
CD40, 4‐1BB	2	2	3
CEACAM5, 4‐1BB	1	2	3
CEACAM5, 4‐1BB	2	1	3
EGFR, CD16a	2	3	3
EGFR, CD3	1	2	3
EGFR, CD3	2	1	3
EGFR, LGR5	2	1	3
EGFR, LGR5	3	2	3
HER2, CD3	1	2	3
HER2, CD3	2	1	3
KLK2, CD3	1	3	3
TGF‐ß, PD‐L1	1	2	3
TGF‐ß, PD‐L1	2	1	3
TIGIT, PD1	1	3	3
4‐1BB, 5 T4	1	1	2
4‐1BB, 5 T4	2	1	2
B7‐H4, 4‐1BB	1	2	2
B7‐H6, CD3	1	2	2
CD47, CLDN18.2	1	2	2
CD47, HER2	1	1	2
CD47, HER2	2	1	2
CD96, TIGIT	1	1	2
CD96, TIGIT	2	1	2
CLDN18.2, CD3	1	1	2
CLDN18.2, CD3	2	1	2
CLDN18.2, CD8	1	1	2
CLDN18.2, CD8	2	1	2
CLDN18.2, PD‐L1	1	1	2
CLDN18.2, PD‐L1	2	1	2
CLDN6, CD3	1	1	2
CLDN6, CD3	2	1	2
DR5, CDH17	1	2	2
EGFR, CD28	2	2	2
ENPP3, CD3	1	2	2
HER2, 4‐1BB	2	2	2
HER3, HER2	2	2	2
HSG, CEACAM5	1	1	2
HSG, CEACAM5	2	1	2
ILT‐4, ILT‐2	1	1	2
ILT‐4, ILT‐2	2	1	2
LAG‐3, CTLA4	1	2	2
PD‐L1, TIGIT	2	2	2
PD‐L1, VEGF	1	2	2
PRAME, CD3	2	1	2
PRAME, CD3	3	1	2
PSMA, CD3	1	1	2
PSMA, CD3	2	1	2
4‐1BB, CLDN18.2	2	1	1
4‐1BB, CLDN4	1	1	1
B7‐H3, CD28	1	1	1
B7‐H3, CD3	2	1	1
CALRmut, CD3	1	1	1
CD20, CD3	2	1	1
CD228, 4‐1BB	1	1	1
CD3	1	1	1
CD3, B7‐H3	1	1	1
CD3, B7‐H4	2	1	1
CD3, CD19	1	1	1
CD3, CD33	1	1	1
CD3, CEACAM5	1	1	1
CD3, CLDN6	1	1	1
CD3, EGFR	1	1	1
CD3, HER2	1	1	1
CD3, MSLN	1	1	1
CD40, MSLN	1	1	1
CD40, PD1	1	1	1
CD47, DLL3	2	1	1
CD71, EGFR	1	1	1
CD73, LAG‐3	1	1	1
CD73, PD1	1	1	1
CDH17, CD3	1	1	1
CDH3, MSLN	1	1	1
CEACAM5, CD3	1	1	1
CEACAM5, DR5	2	1	1
CLDN18.2, CD47	2	1	1
CLDN6, 4‐1BB	1	1	1
DR4, FAP	1	1	1
EGFR, B7‐H3	2	1	1
EGFR, V?9Vd2 T	1	1	1
EpCAM, 4‐1BB	2	1	1
EpCAM, CD40	1	1	1
FAP, 4‐1BB	1	1	1
GPC3, 4‐1BB	1	1	1
HER2, CD47	1	1	1
MSLN, CD3	1	1	1
MSLN, CD40	1	1	1
MSLN, CD47	1	1	1
MUC1, CD16a	1	1	1
MUC16, CD28	2	1	1
OX40, PD‐L1	1	1	1
PD‐L1, GARP	2	1	1
PD‐L1, ILT‐4	1	1	1
PD‐L1, PD‐L2	1	1	1
PD‐L1, TGF‐ß	2	1	1
PD1, ILT‐4	1	1	1
PD1, TGF‐ßR2	2	1	1
TCR ß	2	1	1
TGF‐ß, EGFR	1	1	1
TGF‐ßR2, PD1	1	1	1
TIGIT, LAG‐3	1	1	1
TIGIT, PD‐L1	2	1	1
c‐MET	2	1	1

**FIGURE 5:**
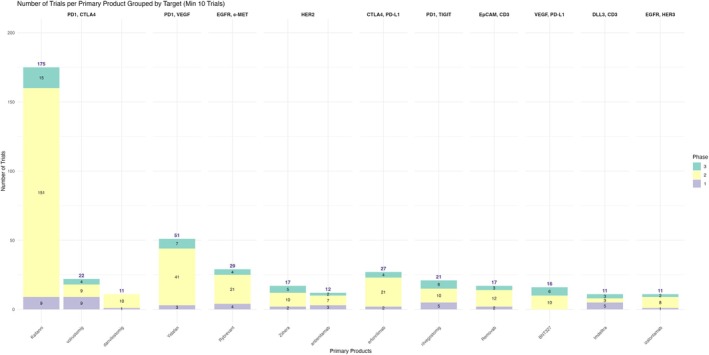
Number of trials per molecule stratified by combination target of interest.

The distribution of clinical molecules also reflects such focus. Dual PD‐1/CTLA‐4 inhibitors (Cadonilimab, Volrustomig, Danviostomig) lead with the highest number of trials (*n* = 208, 30.5%) where Cadonilimab has *n* = 175 trials (25.7%) under progress. Other molecules with more clinical trial investments included Ivonescimab (*n* = 51, 7.5%), targeting PD1‐VEGF, and Amivantamab‐vmjw (*n* = 29, 4.3%), targeting EGFR‐MET. Erfonrilimab and Rilvegostomig were the only molecules being studied in all CTLA‐4/PD‐L1 and PD‐1/TIGIT based trials respectively (Figure 5; Table 4).

**TABLE 4:** Number of BsAbs molecules per target, and number of trials and phases.

Target	Primary products	Phase	Trial count	Product total trials	Target total trials
PD1, CTLA4	Kaitanni	1	9	175	216
PD1, CTLA4	Kaitanni	2	151	175	216
PD1, CTLA4	Kaitanni	3	15	175	216
PD1, CTLA4	Volrustomig	1	9	22	216
PD1, CTLA4	Volrustomig	2	9	22	216
PD1, CTLA4	Volrustomig	3	4	22	216
PD1, CTLA4	Danvilostomig	1	1	11	216
PD1, CTLA4	Danvilostomig	2	10	11	216
PD1, CTLA4	Vudalimab	1	2	8	216
PD1, CTLA4	Vudalimab	2	6	8	216
PD1, VEGF	Yidafan	1	3	51	56
PD1, VEGF	Yidafan	2	41	51	56
PD1, VEGF	Yidafan	3	7	51	56
PD1, VEGF	SSGJ‐707	2	4	4	56
PD1, VEGF	MHB039A	2	1	1	56
EGFR, c‐MET	Rybrevant	1	4	29	36
EGFR, c‐MET	Rybrevant	2	21	29	36
EGFR, c‐MET	Rybrevant	3	4	29	36
EGFR, c‐MET	Amivantamab SC	1	1	4	36
EGFR, c‐MET	Amivantamab SC	2	2	4	36
EGFR, c‐MET	Amivantamab SC	3	1	4	36
EGFR, c‐MET	Bafisontamab	2	3	3	36
HER2	Ziihera	1	2	17	33
HER2	Ziihera	2	10	17	33
HER2	Ziihera	3	5	17	33
HER2	Anbenitamab	1	3	12	33
HER2	Anbenitamab	2	7	12	33
HER2	Anbenitamab	3	2	12	33
HER2	TQB2930	1	1	2	33
HER2	TQB2930	2	1	2	33
HER2	MBS301	1	1	1	33
HER2	XZP‐KM257	1	1	1	33
CTLA4, PD‐L1	Erfonrilimab	1	2	27	27
CTLA4, PD‐L1	Erfonrilimab	2	21	27	27
CTLA4, PD‐L1	Erfonrilimab	3	4	27	27
PD1, TIGIT	Rilvegostomig	1	5	21	26
PD1, TIGIT	Rilvegostomig	2	10	21	26
PD1, TIGIT	rilvegostomig	3	6	21	26
PD1, TIGIT	ZG005	2	5	5	26
VEGF, PD‐L1	BNT327	2	10	16	19
VEGF, PD‐L1	BNT327	3	6	16	19
VEGF, PD‐L1	B1962	1	2	3	19
VEGF, PD‐L1	B1962	2	1	3	19
EpCAM, CD3	Removab	1	2	17	18
EpCAM, CD3	Removab	2	12	17	18
EpCAM, CD3	Removab	3	3	17	18
EpCAM, CD3	A‐337	1	1	1	18
DLL3, CD3	Imdelltra	1	5	11	11
DLL3, CD3	Imdelltra	2	3	11	11
DLL3, CD3	Imdelltra	3	3	11	11
EGFR, HER3	Izalontamab	1	1	11	11
EGFR, HER3	Izalontamab	2	8	11	11
EGFR, HER3	Izalontamab	3	2	11	11
PD‐L1, 4‐1BB	Acasunlimab	1	1	5	10
PD‐L1, 4‐1BB	Acasunlimab	2	3	5	10
PD‐L1, 4‐1BB	Acasunlimab	3	1	5	10
PD‐L1, 4‐1BB	LBL‐024	2	2	2	10
PD‐L1, 4‐1BB	FS222	1	1	1	10
PD‐L1, 4‐1BB	PM1003	2	1	1	10
PD‐L1, 4‐1BB	Ragistomig	1	1	1	10
CD3, DLL3	Obrixtamig	1	6	8	9
CD3, DLL3	Obrixtamig	2	2	8	9
c‐MET, EGFR	PM1080	1	2	3	9
c‐MET, EGFR	PM1080	3	1	3	9
c‐MET, EGFR	Pamvatamig	1	1	3	9
c‐MET, EGFR	Pamvatamig	2	2	3	9
c‐MET, EGFR	CKD‐702	1	1	2	9
c‐MET, EGFR	CKD‐702	2	1	2	9
c‐MET, EGFR	FPI‐2053	1	1	1	9
CD3, DLL3	QLS31904	1	1	1	9
PD1, LAG‐3	Tebotelimab	1	2	6	8
PD1, LAG‐3	Tebotelimab	2	2	6	8
PD1, LAG‐3	Tebotelimab	3	2	6	8
PD1, LAG‐3	AK129	2	1	1	8
PD1, LAG‐3	EMB‐02	2	1	1	8
ANG2, VEGF	BI 836880	1	5	7	7
ANG2, VEGF	BI 836880	2	2	7	7
PD‐L1, PD1	Reozalimab	1	5	6	7
PD‐L1, PD1	Reozalimab	2	1	6	7
PD‐L1, PD1	SSGJ‐706	1	1	1	7
VEGF, DLL4	CTX‐009	2	5	6	6
VEGF, DLL4	CTX‐009	3	1	6	6
PD1, TIM‐3	Sabestomig	1	3	5	6
PD1, TIM‐3	Sabestomig	2	2	5	6
VEGF, PD1	RC148	1	1	2	6
VEGF, PD1	RC148	2	1	2	6
PD1, TIM‐3	LB1410	2	1	1	6
VEGF, PD1	AI‐081	2	1	1	6
VEGF, PD1	JS207	1	1	1	6
VEGF, PD1	LM‐299	2	1	1	6
VEGF, PD1	SCTB14	2	1	1	6
CTLA4, PD1	Lorigerlimab	1	2	5	5
CTLA4, PD1	Lorigerlimab	2	3	5	5
DLL4, VEGF	Navicixizumab	1	3	5	5
DLL4, VEGF	Navicixizumab	2	1	5	5
DLL4, VEGF	Navicixizumab	3	1	5	5
CD3, EpCAM	M701	1	1	4	5
CD3, EpCAM	M701	2	2	4	5
CD3, EpCAM	M701	3	1	4	5
TIGIT, PVRIG	PM1009	1	1	2	5
TIGIT, PVRIG	PM1009	2	1	2	5
TIGIT, PVRIG	SHR‐2002	1	2	2	5
CD3, EpCAM	BA3182	1	1	1	5
TIGIT, PVRIG	SIM0348	1	1	1	5
CD3, STEAP1	Xaluritamig	1	3	4	4
CD3, STEAP1	Xaluritamig	3	1	4	4
PD1, CD47	HX‐009	1	2	4	4
PD1, CD47	HX‐009	2	2	4	4
CD28, PSMA	Nezastomig	2	3	3	4
CD3, CLDN18.2	QLS31905	1	1	3	4
CD3, CLDN18.2	QLS31905	2	2	3	4
CD3, MUC16	ubamatamab	2	3	3	4
CD3, PSMA	CC‐1	1	2	3	4
CD3, PSMA	CC‐1	2	1	3	4
CD47, PD‐L1	Simridarlimab	1	2	3	4
CD47, PD‐L1	Simridarlimab	2	1	3	4
PD‐L1, CD47	6 MW3211	2	3	3	4
4‐1BB, PD‐L1	QL301	2	2	2	4
HER2, PD1	SSGJ‐705	1	2	2	4
HER2, PD1	Fidasimtamab	1	1	2	4
HER2, PD1	Fidasimtamab	2	1	2	4
PD‐L1, LAG‐3	IBI‐323	1	1	2	4
PD‐L1, LAG‐3	IBI‐323	2	1	2	4
4‐1BB, PD‐L1	AP203	2	1	1	4
4‐1BB, PD‐L1	BH3120	1	1	1	4
CD28, PSMA	JNJ‐9401	1	1	1	4
CD3, CLDN18.2	ASP2138	1	1	1	4
CD3, MUC16	LBL‐033	2	1	1	4
CD3, PSMA	JNJ‐8114	1	1	1	4
CD47, PD‐L1	IMM2520	1	1	1	4
PD‐L1, CD47	LB101	2	1	1	4
PD‐L1, LAG‐3	ABL501	1	1	1	4
PD‐L1, LAG‐3	FS118	2	1	1	4
4‐1BB, EGFR	HLX35	1	3	3	3
CD39, TGF‐ß	ES014	1	2	3	3
CD39, TGF‐ß	ES014	2	1	3	3
CD40, 4‐1BB	GEN1042	1	1	3	3
CD40, 4‐1BB	GEN1042	2	2	3	3
EGFR, CD16a	AFM24	2	3	3	3
EGFR, LGR5	Petosemtamab	2	1	3	3
EGFR, LGR5	Petosemtamab	3	2	3	3
KLK2, CD3	JNJ‐8343	1	3	3	3
TIGIT, PD1	IBI321	1	3	3	3
CD3, GPC3	ERY974	1	2	2	3
CEACAM5, 4‐1BB	BGB‐B167	1	2	2	3
EGFR, CD3	TAK‐186	1	1	2	3
EGFR, CD3	TAK‐186	2	1	2	3
HER2, CD3	ISB 1302	1	1	2	3
HER2, CD3	ISB 1302	2	1	2	3
TGF‐ß, PD‐L1	Y101D	1	1	2	3
TGF‐ß, PD‐L1	Y101D	2	1	2	3
CD3, GPC3	CM350	2	1	1	3
CEACAM5, 4‐1BB	LM‐24C5	2	1	1	3
EGFR, CD3	SMET‐12	1	1	1	3
HER2, CD3	Runimotamab	1	1	1	3
TGF‐ß, PD‐L1	PM8001	1	1	1	3
B7‐H6, CD3	BI 765049	1	2	2	2
CD47, HER2	IMM2902	1	1	2	2
CD47, HER2	IMM2902	2	1	2	2
CD96, TIGIT	AGEN1777	1	1	2	2
CD96, TIGIT	AGEN1777	2	1	2	2
CLDN18.2, CD8	LB4330	1	1	2	2
CLDN18.2, CD8	LB4330	2	1	2	2
CLDN18.2, PD‐L1	Q‐1802	1	1	2	2
CLDN18.2, PD‐L1	Q‐1802	2	1	2	2
DR5, CDH17	BI 905711	1	2	2	2
EGFR, CD28	Dalmitamig	2	2	2	2
HER3, HER2	Bizengri	2	2	2	2
HSG, CEACAM5	TF2	1	1	2	2
HSG, CEACAM5	TF2	2	1	2	2
LAG‐3, CTLA4	Bavunalimab	1	2	2	2
PRAME, CD3	Brenetafusp	2	1	2	2
PRAME, CD3	Brenetafusp	3	1	2	2
4‐1BB, 5 T4	ALG.APV‐527	2	1	1	2
4‐1BB, 5 T4	FTL008.16	1	1	1	2
B7‐H4, 4‐1BB	ABL103	1	1	1	2
B7‐H4, 4‐1BB	HBM7008	1	1	1	2
CD47, CLDN18.2	AK132	1	1	1	2
CD47, CLDN18.2	SG1906	1	1	1	2
CLDN18.2, CD3	AZD5863	2	1	1	2
CLDN18.2, CD3	IBI‐389	1	1	1	2
CLDN6, CD3	BNT142	2	1	1	2
CLDN6, CD3	XmAb541	1	1	1	2
ENPP3, CD3	JNJ‐0387	1	1	1	2
ENPP3, CD3	XmAb819	1	1	1	2
HER2, 4‐1BB	ABL105	2	1	1	2
HER2, 4‐1BB	AP 402	2	1	1	2
ILT‐4, ILT‐2	NGM707	2	1	1	2
ILT‐4, ILT‐2	PF‐07826390	1	1	1	2
PD‐L1, TIGIT	HB0036	2	1	1	2
PD‐L1, TIGIT	PM1022	2	1	1	2
PD‐L1, VEGF	CVL‐006	1	1	1	2
PD‐L1, VEGF	SYN‐2510	1	1	1	2
PSMA, CD3	CCW702	1	1	1	2
PSMA, CD3	REGN4336	2	1	1	2
4‐1BB, CLDN18.2	PM1032	2	1	1	1
4‐1BB, CLDN4	ASP1002	1	1	1	1
B7‐H3, CD28	XmAb808	1	1	1	1
B7‐H3, CD3	TAK‐280	2	1	1	1
c‐MET	Davutamig	2	1	1	1
CALRmut, CD3	JNJ‐9968	1	1	1	1
CD20, CD3	Epkinly	2	1	1	1
CD228, 4‐1BB	PF‐08046049	1	1	1	1
CD3	TGI‐6	1	1	1	1
CD3, B7‐H3	CC‐3	1	1	1	1
CD3, B7‐H4	GEN1047	2	1	1	1
CD3, CD19	Blincyto	1	1	1	1
CD3, CD33	Vixtimotamab	1	1	1	1
CD3, CEACAM5	BA1202	1	1	1	1
CD3, CLDN6	CTIM‐76	1	1	1	1
CD3, EGFR	CX‐904	1	1	1	1
CD3, HER2	M802	1	1	1	1
CD3, MSLN	ZW171	1	1	1	1
CD40, MSLN	Inezetamab	1	1	1	1
CD40, PD1	YH008	1	1	1	1
CD47, DLL3	PT217	2	1	1	1
CD71, EGFR	KK2260	1	1	1	1
CD73, LAG‐3	AK137	1	1	1	1
CD73, PD1	AK131	1	1	1	1
CDH17, CD3	Cabotamig	1	1	1	1
CDH3, MSLN	AMG 305	1	1	1	1
CEACAM5, CD3	NILK‐2301	1	1	1	1
CEACAM5, DR5	IBI3004	2	1	1	1
CLDN18.2, CD47	PT886	2	1	1	1
CLDN6, 4‐1BB	NBL‐028	1	1	1	1
DR4, FAP	GEN1057	1	1	1	1
EGFR, B7‐H3	IBI‐334	2	1	1	1
EGFR, V?9Vd2 T	PF‐08046052	1	1	1	1
EpCAM, 4‐1BB	BNT314	2	1	1	1
EpCAM, CD40	KK2269	1	1	1	1
FAP, 4‐1BB	BI 765179	1	1	1	1
GPC3, 4‐1BB	BGB‐B2033	1	1	1	1
HER2, CD47	D3L‐001	1	1	1	1
MSLN, CD3	JNJ‐2421	1	1	1	1
MSLN, CD40	ABBV‐428	1	1	1	1
MSLN, CD47	NI‐1801	1	1	1	1
MUC1, CD16a	BGB‐B3227	1	1	1	1
MUC16, CD28	REGN5668	2	1	1	1
OX40, PD‐L1	EMB‐09	1	1	1	1
PD‐L1, GARP	BPB‐101	2	1	1	1
PD‐L1, ILT‐4	SPX‐303	1	1	1	1
PD‐L1, PD‐L2	IMGS‐001	1	1	1	1
PD‐L1, TGF‐ß	HB0028	2	1	1	1
PD1, ILT‐4	CDX‐585	1	1	1	1
PD1, TGF‐ßR2	LBL‐015	2	1	1	1
TCR ß	Invikafusp alfa	2	1	1	1
TGF‐ß, EGFR	Ficerafusp alfa	1	1	1	1
TGF‐ßR2, PD1	INCA33890	1	1	1	1
TIGIT, LAG‐3	ZGGS15	1	1	1	1
TIGIT, PD‐L1	HLX301	2	1	1	1

COMPASSION‐15, NCT050008783, is a phase III trial of first‐line cadonilimab plus oxaliplatin‐capecitabine versus chemotherapy alone. It randomized 610 patients with locally advanced or metastatic HER‐2 negative gastric or gastroesophageal adenocarcinoma. The cadonilimab group *n* = 305 achieved significantly prolonged overall survival (OS) 14.1 versus 11.1 months, hazard ratio (HR) 0.66 (95% CI 0.54–0.81; *p* < 0.001) [9]. Median progression‐free survival (mPFS) was longer in the cadonilimab group, 7 versus 5.3 months, HR 0.53 (95% CI 0.44–0.65), and overall response rate (ORR) of 65.2% versus 48.9%. Although treatment‐related adverse events were higher in the cadonilimab group of 65.9% versus 53.6%, they were similar to previous trials investigating anti‐PD‐L1 plus chemotherapy [9]. A combination of cadonilimab and platinum‐based chemotherapy with or without bevacizumab showed improved PFS as first‐line treatment in patients with advanced or metastatic cervical cancer—COMPASSION‐16, NCT04982237 [10]. A total of 445 patients were randomized; 222 in the cadonilimab group achieved prolonged PFS compared to placebo 12.7 versus 8.1 months, HR 0.62 (95% CI 0.49–0.80; *p* < 0.0001) and improved 24 months OS 62.2% versus 48.4% [10].

**FIGURE 6:**
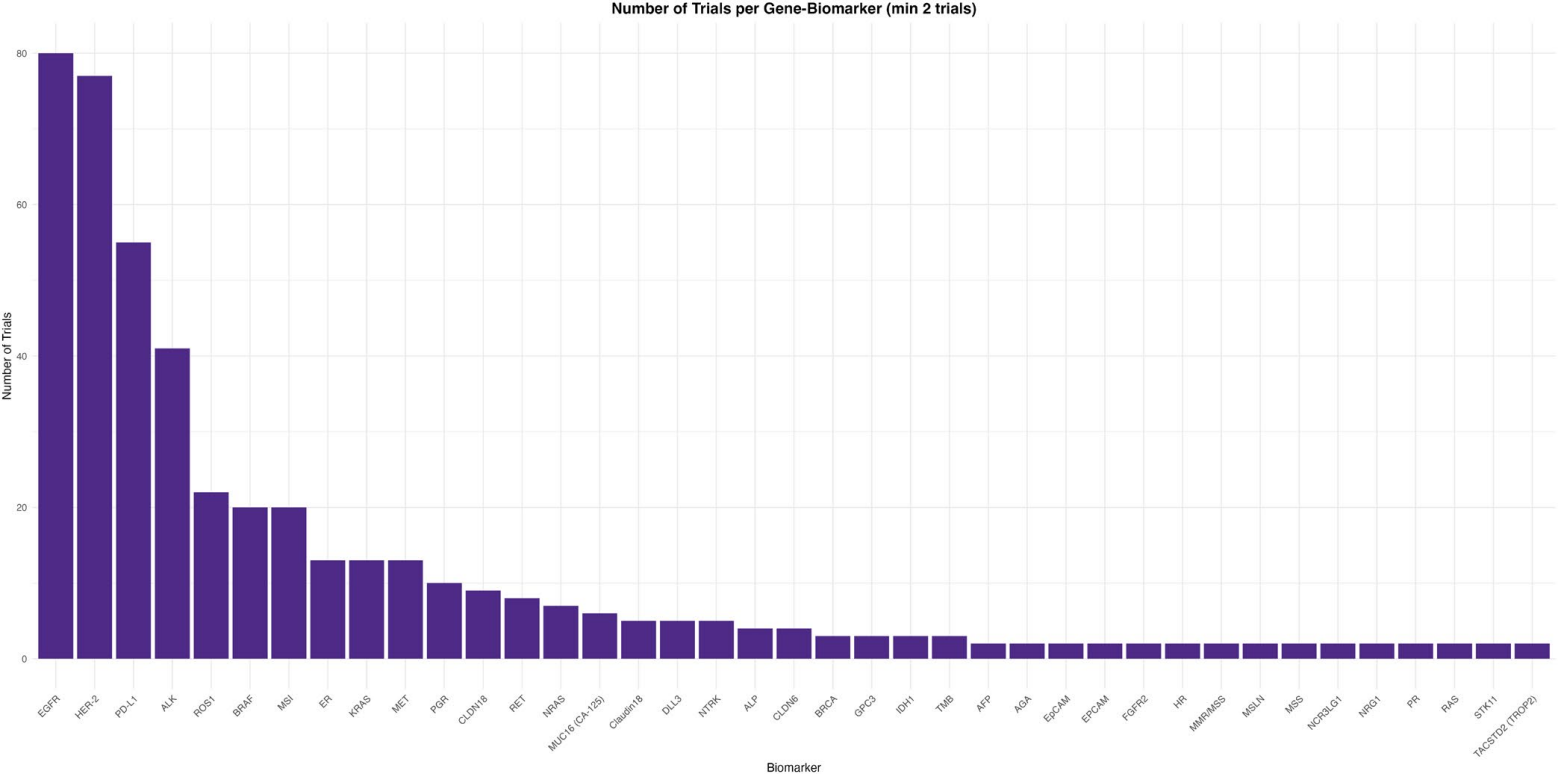
Number of trials per gene biomarker.

We sought to specifically evaluate the biomarker requirements, along with a focus on genetic alterations, to better understand the role of precision oncology in the development of these molecules. Only 38% of trials, *n* = 258, specified a biomarker in its inclusion criteria. While there were 66 different biomarkers included among the trials, *EGFR*, *HER2* and PD‐L1 were represented in 82% of trials, *n* = 212. Among those specified 258 trials, *EGFR* led the highest prevalence (*n* = 80, 31%) followed by *HER2* (*n* = 77, 29.8%), PD‐L1 (*n* = 55, 21.3%) and *ALK* (*n* = 41, 15.8%). Other promising biomarkers included *BRAF*, MSI included in *n* = 20 trials, 7.7% each and *ER*, *KRAS* and *MET* included in *n* = 13 trials, 5% each (Figure 6; Table 5).

**TABLE 5:** Number of trials for gene biomarker.

Biomarker	NCT count
EGFR	80
HER‐2	77
PD‐L1	55
ALK	41
ROS1	22
BRAF	20
MSI	20
ER	13
KRAS	13
MET	13
PGR	10
CLDN18	9
RET	8
NRAS	7
MUC16 (CA‐125)	6
Claudin18	5
DLL3	5
NTRK	5
ALP	4
CLDN6	4
BRCA	3
GPC3	3
IDH1	3
TMB	3
AFP	2
AGA	2
EPCAM	2
EpCAM	2
FGFR2	2
HR	2
MMR/MSS	2
MSLN	2
MSS	2
NCR3LG1	2
NRG1	2
PR	2
RAS	2
STK11	2
TACSTD2 (TROP2)	2
MET	1
CD4	1
CDH17	1
CDKN2A (p16)	1
CEACAM5	1
CTLA‐4	1
FGFR	1
FOLH1	1
HLA‐A	1
IL‐6	1
KIT	1
LAG3	1
MLH1	1
MMR	1
MSH2	1
MSH6	1
MUC1	1
MUC16	1
NCR3LG1 (B7‐H6)	1
NTRK1	1
NTRK2	1
NTRK3	1
PD L1	1
PD‐1	1
PMS2	1
SMARCB1 negative	1
TFE3	1

**FIGURE 7:**
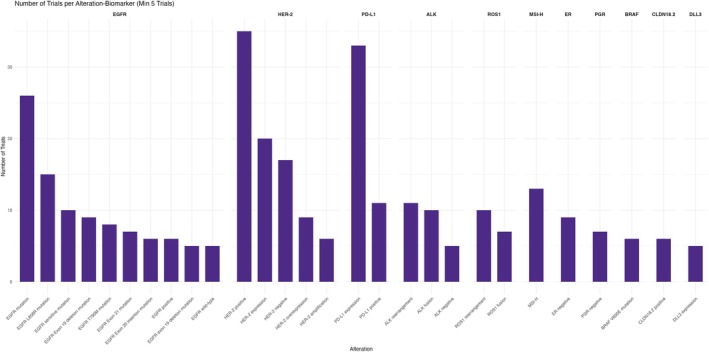
Number of trials per gene alteration stratified by gene biomarker.

**FIGURE 8:**
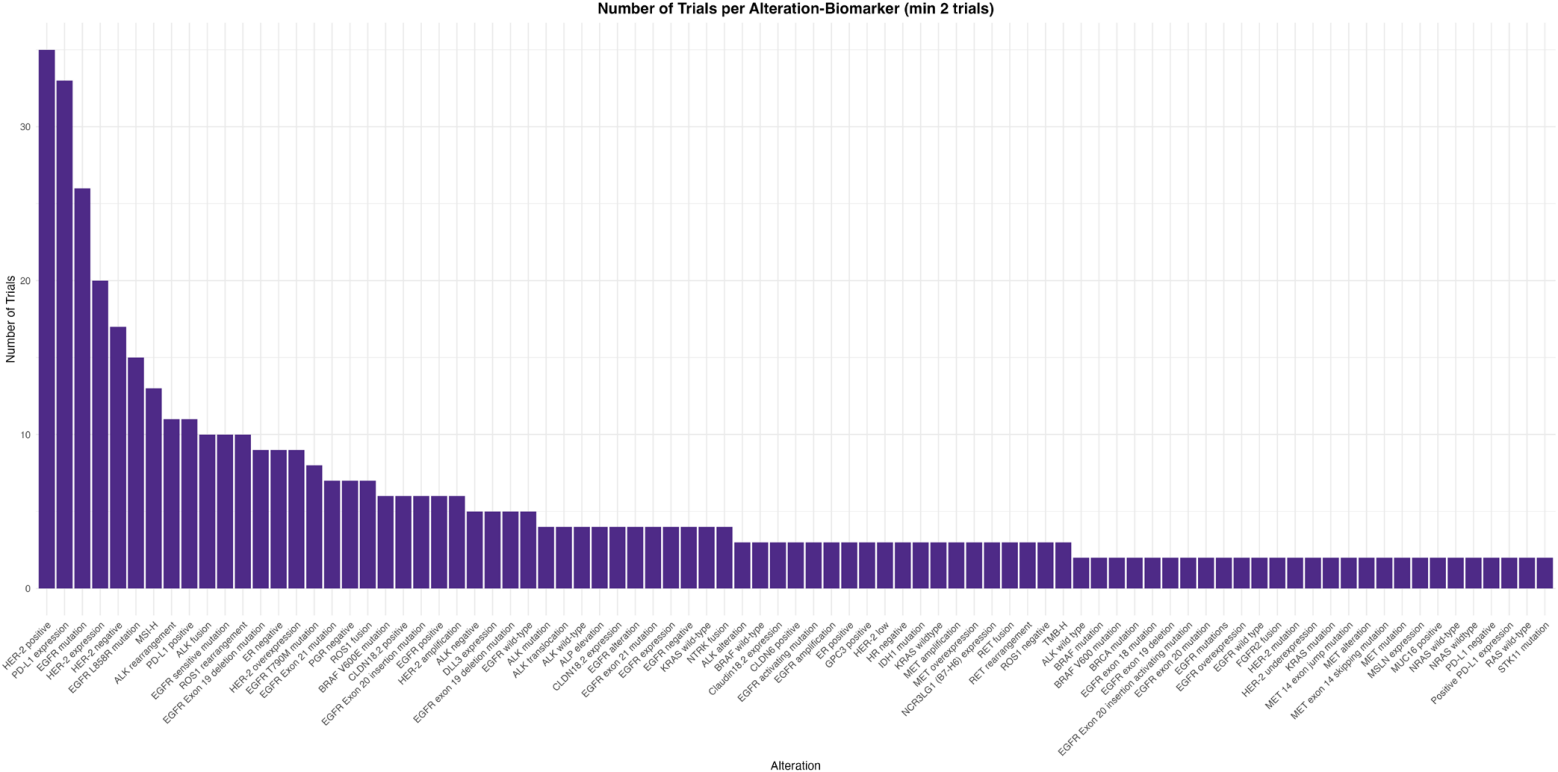
Number of trials per specific gene biomarker alteration.

Among all gene alterations that preserved interest among researchers, *EGFR* and *HER2* all‐type alterations were included in *n* = 149 trials and *n* = 97 trials, respectively. Gene‐specific alterations were also represented among potential BsAbs targets. *HER2*‐positive alterations were represented in *n* = 35 trials, followed by *EGFR* mutations in *n* = 26 trials (Figures 7 and 8; Tables 6 and 7).

**TABLE 6:** Number of trials per gene alteration stratified by gene biomarker.

Alteration	Gene	Alteration total trials	Gene total trials
EGFR Exon 19 deletion	EGFR	1	149
EGFR Exon 19 deletion mutation	EGFR	9	149
EGFR Exon 20 insertion activating mutation	EGFR	2	149
EGFR Exon 20 insertion mutation	EGFR	6	149
EGFR Exon 20 mutation	EGFR	1	149
EGFR Exon 21 mutation	EGFR	7	149
EGFR G719X mutation	EGFR	1	149
EGFR L858R activating mutation	EGFR	1	149
EGFR L858R mutation	EGFR	15	149
EGFR L861Q mutation	EGFR	1	149
EGFR Positve	EGFR	1	149
EGFR S768I mutation	EGFR	1	149
EGFR Somatic activating mutation	EGFR	1	149
EGFR T790M mutation	EGFR	8	149
EGFR T790M negative mutation	EGFR	1	149
EGFR T790M positive mutation	EGFR	1	149
EGFR Thr790Met negative mutation	EGFR	1	149
EGFR activating mutation	EGFR	3	149
EGFR alteration	EGFR	4	149
EGFR amplification	EGFR	3	149
EGFR exon 18 mutation	EGFR	2	149
EGFR exon 19 deletion	EGFR	2	149
EGFR exon 19 deletion mutation	EGFR	5	149
EGFR exon 20 insertion mutation	EGFR	1	149
EGFR exon 20 mutation	EGFR	2	149
EGFR exon 21 mutation	EGFR	4	149
EGFR expression	EGFR	4	149
EGFR mutantion	EGFR	1	149
EGFR mutation	EGFR	26	149
EGFR mutations	EGFR	2	149
EGFR negative	EGFR	4	149
EGFR overexpression	EGFR	2	149
EGFR positive	EGFR	6	149
EGFR rearrangement	EGFR	1	149
EGFR sensitive mutation	EGFR	10	149
EGFR sensitizing mutation	EGFR	1	149
EGFR wild type	EGFR	2	149
EGFR wild‐type	EGFR	5	149
EGFR wildtype	EGFR	1	149
HER‐2 amplification	HER‐2	6	97
HER‐2 copy number	HER‐2	1	97
HER‐2 copy number alteration	HER‐2	1	97
HER‐2 copy number alterations	HER‐2	1	97
HER‐2 expression	HER‐2	20	97
HER‐2 low	HER‐2	3	97
HER‐2 mutation	HER‐2	2	97
HER‐2 negative	HER‐2	17	97
HER‐2 overexpression	HER‐2	9	97
HER‐2 positive	HER‐2	35	97
HER‐2 underexpression	HER‐2	2	97
ALK Positve	ALK	1	47
ALK alteration	ALK	3	47
ALK fusion	ALK	10	47
ALK fusions	ALK	1	47
ALK mutation	ALK	4	47
ALK negative	ALK	5	47
ALK negative fusion	ALK	1	47
ALK rearrangement	ALK	11	47
ALK translocation	ALK	4	47
ALK wild type	ALK	2	47
ALK wild‐type	ALK	4	47
ALK wildtype	ALK	1	47
PD‐L1 expression	PD‐L1	33	47
PD‐L1 high	PD‐L1	1	47
PD‐L1 negative	PD‐L1	2	47
PD‐L1 positive	PD‐L1	11	47
ROS1 Positve	ROS1	1	26
ROS1 alteration	ROS1	1	26
ROS1 fusion	ROS1	7	26
ROS1 fusion negative	ROS1	1	26
ROS1 mutation	ROS1	1	26
ROS1 negative	ROS1	3	26
ROS1 negative fusion	ROS1	1	26
ROS1 rearrangement	ROS1	10	26
ROS1 wild type	ROS1	1	26
MET 14 exon jump mutation	MET	2	18
MET Exon 14 skipping alteration	MET	1	18
MET aberration	MET	1	18
MET alteration	MET	2	18
MET amplification	MET	3	18
MET exon 14 skipping mutation	MET	2	18
MET expression	MET	1	18
MET mutation	MET	2	18
MET overexpression	MET	3	18
MET positive	MET	1	18
BRAF V600 mutation	BRAF	2	17
BRAF V600E	BRAF	1	17
BRAF V600E mutation	BRAF	6	17
BRAF WT	BRAF	1	17
BRAF mutation	BRAF	2	17
BRAF wild type	BRAF	1	17
BRAF wild‐type	BRAF	3	17
BRAF wildtype	BRAF	1	17
ER expression	ER	1	13
ER negative	ER	9	13
ER positive	ER	3	13
KRAS G12C negative mutation	KRAS	1	13
KRAS G12D negative mutation	KRAS	1	13
KRAS G12V negative mutation	KRAS	1	13
KRAS mutation	KRAS	2	13
KRAS wild type	KRAS	1	13
KRAS wild‐type	KRAS	4	13
KRAS wildtype	KRAS	3	13
MSI‐H	MSI‐H	13	13
CLDN18.2 expression	CLDN18.2	4	10
CLDN18.2 positive	CLDN18.2	6	10
PGR expression	PGR	1	9
PGR negative	PGR	7	9
PGR positive	PGR	1	9
RET fusion	RET	3	7
RET negative fusion	RET	1	7
RET rearrangement	RET	3	7
NRAS mutation	NRAS	1	6
NRAS wild type	NRAS	1	6
NRAS wild‐type	NRAS	2	6
NRAS wildtype	NRAS	2	6
RAS WT	RAS	1	6
RAS mutation	RAS	1	6
RAS wild type	RAS	1	6
RAS wild‐type	RAS	2	6
RAS wildtype	RAS	1	6
DLL3 expression	DLL3	5	5
ALP elevation	ALP	4	4
CLDN6 expression	CLDN6	1	4
CLDN6 positive	CLDN6	3	4
Claudin18.2 expression	Claudin18.2	3	4
Claudin18.2 overexpression	Claudin18.2	1	4
HR negative	HR	3	4
HR positive	HR	1	4
NCR3LG1 (B7‐H6) expression	NCR3LG1	3	4
NCR3LG1 (B7‐H6) positive	NCR3LG1	1	4
NTRK fusion	NTRK	4	4
BRCA germline mutation	BRCA	1	3
BRCA mutation	BRCA	2	3
GPC3 positive	GPC3	3	3
IDH1 mutation	IDH1	3	3
MSLN expression	MSLN	2	3
MSLN positive	MSLN	1	3
MUC16 expression	MUC16	1	3
MUC16 positive	MUC16	2	3
TMB‐H	TMB‐H	3	3
CDKN2A negative	CDKN2A	1	2
CDKN2A positive	CDKN2A	1	2
EGFR‐sensitive mutation	EGFR‐sensitive	1	2
EGFR‐sensitive mutations	EGFR‐sensitive	1	2
EPCAM expression	EPCAM	1	2
EPCAM positive	EPCAM	1	2
EpCAM overexpression	EpCAM	1	2
EpCAM positive	EpCAM	1	2
FGFR2 fusion	FGFR2	2	2
KIT amplification	KIT	1	2
KIT mutation	KIT	1	2
NRG1 fusion	NRG1	1	2
NRG1 gene fusion	NRG1	1	2
PR negative	PR	1	2
PR positive	PR	1	2
Positive PD‐L1 expression	Positive	2	2
SMARCB1 loss	SMARCB1	1	2
SMARCB1 negative	SMARCB1	1	2
STK11 mutation	STK11	2	2
negative BRAF V600E mutation	negative	1	2
negative EGFR sensitive mutations	negative	1	2
AFP positive	AFP	1	1
AGA negative	AGA	1	1
AGA‐negative	AGA‐negative	1	1
CD4 positive	CD4	1	1
CDH17 expression	CDH17	1	1
CEACAM5 overexpression	CEACAM5	1	1
CLDN18.2‐positive	CLDN18.2‐positive	1	1
Claudin18.2‐positive	Claudin18.2‐positive	1	1
FGFR mutation	FGFR	1	1
FOLH1 (PSMA) expression	FOLH1	1	1
HLA‐A*02:01 positive	HLA‐A*02:01	1	1
Hormone receptor positive	Hormone	1	1
LAG3 expression	LAG3	1	1
Loss of MMR protein expression	Loss	1	1
MLH1 expression	MLH1	1	1
MMR expression	MMR	1	1
MMRD (mismatch repair deficient)	MMRD	1	1
MSH2 expression	MSH2	1	1
MSH6 expression	MSH6	1	1
MSI low	MSI	1	1
MSI‐low	MSI‐low	1	1
MUC1 expression	MUC1	1	1
NTRK1 fusion	NTRK1	1	1
NTRK2 fusion	NTRK2	1	1
NTRK3 fusion	NTRK3	1	1
PD L1 expression	PD	1	1
PD‐1 expression	PD‐1	1	1
PMS2 expression	PMS2	1	1
RAF wild‐type	RAF	1	1
TFE3 rearrangement	TFE3	1	1
cMet Exon 14 skipping mutation	cMet	1	1

**TABLE 7:** Number of trials per specific gene biomarker alteration.

Alteration	NCT count
HER‐2 positive	35
PD‐L1 expression	33
EGFR mutation	26
HER‐2 expression	20
HER‐2 negative	17
EGFR L858R mutation	15
MSI‐H	13
ALK rearrangement	11
PD‐L1 positive	11
ALK fusion	10
EGFR sensitive mutation	10
ROS1 rearrangement	10
EGFR Exon 19 deletion mutation	9
ER negative	9
HER‐2 overexpression	9
EGFR T790M mutation	8
EGFR Exon 21 mutation	7
PGR negative	7
ROS1 fusion	7
BRAF V600E mutation	6
CLDN18.2 positive	6
EGFR Exon 20 insertion mutation	6
EGFR positive	6
HER‐2 amplification	6
ALK negative	5
DLL3 expression	5
EGFR exon 19 deletion mutation	5
EGFR wild‐type	5
ALK mutation	4
ALK translocation	4
ALK wild‐type	4
ALP elevation	4
CLDN18.2 expression	4
EGFR alteration	4
EGFR exon 21 mutation	4
EGFR expression	4
EGFR negative	4
KRAS wild‐type	4
NTRK fusion	4
ALK alteration	3
BRAF wild‐type	3
CLDN6 positive	3
Claudin18.2 expression	3
EGFR activating mutation	3
EGFR amplification	3
ER positive	3
GPC3 positive	3
HER‐2 low	3
HR negative	3
IDH1 mutation	3
KRAS wildtype	3
MET amplification	3
MET overexpression	3
NCR3LG1 (B7‐H6) expression	3
RET fusion	3
RET rearrangement	3
ROS1 negative	3
TMB‐H	3
ALK wild type	2
BRAF V600 mutation	2
BRAF mutation	2
BRCA mutation	2
EGFR Exon 20 insertion activating mutation	2
EGFR exon 18 mutation	2
EGFR exon 19 deletion	2
EGFR exon 20 mutation	2
EGFR mutations	2
EGFR overexpression	2
EGFR wild type	2
FGFR2 fusion	2
HER‐2 mutation	2
HER‐2 underexpression	2
KRAS mutation	2
MET 14 exon jump mutation	2
MET alteration	2
MET exon 14 skipping mutation	2
MET mutation	2
MSLN expression	2
MUC16 positive	2
NRAS wild‐type	2
NRAS wildtype	2
PD‐L1 negative	2
Positive PD‐L1 expression	2
RAS wild‐type	2
STK11 mutation	2
AFP positive	1
AGA negative	1
AGA‐negative	1
ALK Positve	1
ALK fusions	1
ALK negative fusion	1
ALK wildtype	1
BRAF V600E	1
BRAF WT	1
BRAF wild type	1
BRAF wildtype	1
BRCA germline mutation	1
CD4 positive	1
CDH17 expression	1
CDKN2A negative	1
CDKN2A positive	1
CEACAM5 overexpression	1
CLDN18.2‐positive	1
CLDN6 expression	1
Claudin18.2 overexpression	1
Claudin18.2‐positive	1
EGFR Exon 19 deletion	1
EGFR Exon 20 mutation	1
EGFR G719X mutation	1
EGFR L858R activating mutation	1
EGFR L861Q mutation	1
EGFR Positve	1
EGFR S768I mutation	1
EGFR Somatic activating mutation	1
EGFR T790M negative mutation	1
EGFR T790M positive mutation	1
EGFR Thr790Met negative mutation	1
EGFR exon 20 insertion mutation	1
EGFR mutantion	1
EGFR rearrangement	1
EGFR sensitizing mutation	1
EGFR wildtype	1
EGFR‐sensitive mutation	1
EGFR‐sensitive mutations	1
EPCAM expression	1
EPCAM positive	1
ER expression	1
EpCAM overexpression	1
EpCAM positive	1
FGFR mutation	1
FOLH1 (PSMA) expression	1
HER‐2 copy number	1
HER‐2 copy number alteration	1
HER‐2 copy number alterations	1
HLA‐A*02:01 positive	1
HR positive	1
Hormone receptor positive	1
KIT amplification	1
KIT mutation	1
KRAS G12C negative mutation	1
KRAS G12D negative mutation	1
KRAS G12V negative mutation	1
KRAS wild type	1
LAG3 expression	1
Loss of MMR protein expression	1
MET Exon 14 skipping alteration	1
MET aberration	1
MET expression	1
MET positive	1
MLH1 expression	1
MMR expression	1
MMRD (mismatch repair deficient)	1
MSH2 expression	1
MSH6 expression	1
MSI low	1
MSI‐low	1
MSLN positive	1
MUC1 expression	1
MUC16 expression	1
NCR3LG1 (B7‐H6) positive	1
NRAS mutation	1
NRAS wild type	1
NRG1 fusion	1
NRG1 gene fusion	1
NTRK1 fusion	1
NTRK2 fusion	1
NTRK3 fusion	1
PD L1 expression	1
PD‐1 expression	1
PD‐L1 high	1
PGR expression	1
PGR positive	1
PMS2 expression	1
PR negative	1
PR positive	1
RAF wild‐type	1
RAS WT	1
RAS mutation	1
RAS wild type	1
RAS wildtype	1
RET negative fusion	1
ROS1 Positve	1
ROS1 alteration	1
ROS1 fusion negative	1
ROS1 mutation	1
ROS1 negative fusion	1
ROS1 wild type	1
SMARCB1 loss	1
SMARCB1 negative	1
TFE3 rearrangement	1
cMet Exon 14 skipping mutation	1
Negative BRAF V600E mutation	1
Negative EGFR sensitive mutations	1

### 3.3 Trial Phase and Sponsors

**FIGURE 9:**
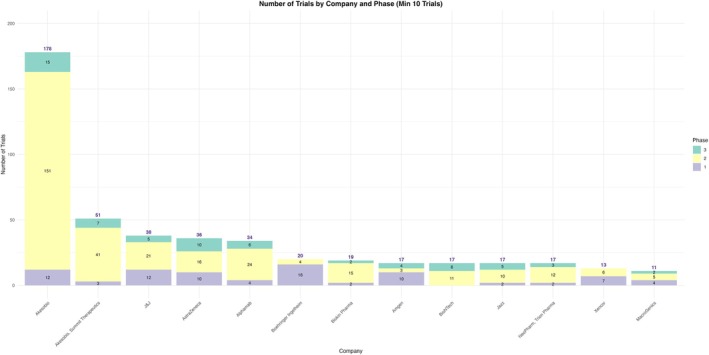
Number of trials per biopharmaceutical company and phases.

We found that BsAbs development was primarily driven by biotechnology companies (Akesobio, Alphamab, Biokin Pharma, etc.) compared to large pharmaceutical companies (J&J, AstraZeneca, and Boehringer Ingelheim). Akesobio dominated the research development of BsAbs with 178 trials, (26%), where 151 trials (22.2%) were within phase 2. Its collaboration with Summit Therapeutics brought 51 more trials to their BsAbs research contribution which reflects the promising nature of BsAbs development partnerships especially among biotechnology companies. Other competitive biotechnology companies like Alphamab and Biokin Pharma had a comparable number of BsAbs clinical trials when compared to large multinational pharmaceutical companies like J&J, AstraZeneca and Boehringer. Interestingly, 196 trials (28.8%) distributed among all pharmaceutical companies were within phase 1, which underscores the innovative but nascent state of these therapies (Figure 9; Table 8). With that broad distribution of the registered clinical trials among pharmaceutical companies, 360 trials (53%) are actively recruiting,130 trials (19%) yet to recruit but only 29 trials (4.3%) were terminated, which reflects the promising future of BsAbs molecules. (Table 9).

**TABLE 8:** Number of trials per biopharmaceutical company and phase.

Primary_Institutions	Phase	Trial count	Total trials
Akesobio	1	12	178
Akesobio	2	151	178
Akesobio	3	15	178
Akesobio, Summit Therapeutics	1	3	51
Akesobio, Summit Therapeutics	2	41	51
Akesobio, Summit Therapeutics	3	7	51
J&J	1	12	38
J&J	2	21	38
J&J	3	5	38
AstraZeneca	1	10	36
AstraZeneca	2	16	36
AstraZeneca	3	10	36
Alphamab	1	4	34
Alphamab	2	24	34
Alphamab	3	6	34
Boehringer Ingelheim	1	16	20
Boehringer Ingelheim	2	4	20
Biokin Pharma	1	2	19
Biokin Pharma	2	15	19
Biokin Pharma	3	2	19
Amgen	1	10	17
Amgen	2	3	17
Amgen	3	4	17
BioNTech	2	11	17
BioNTech	3	6	17
Jazz	1	2	17
Jazz	2	10	17
Jazz	3	5	17
NeoPharm, Trion Pharma	1	2	17
NeoPharm, Trion Pharma	2	12	17
NeoPharm, Trion Pharma	3	3	17
Xencor	1	7	13
Xencor	2	6	13
MacroGenics	1	4	11
MacroGenics	2	5	11
MacroGenics	3	2	11
Biotheus	1	4	9
Biotheus	2	4	9
Biotheus	3	1	9
Regeneron	2	9	9
Innovent Biologics	1	4	8
Innovent Biologics	2	4	8
3SBio	1	3	7
3SBio	2	4	7
Genmab	1	2	7
Genmab	2	4	7
Genmab	3	1	7
YZY Biopharma	1	3	7
YZY Biopharma	2	3	7
YZY Biopharma	3	1	7
ABL Bio, Compass Therap	2	5	6
ABL Bio, Compass Therap	3	1	6
Eli Lilly	1	5	6
Eli Lilly	2	1	6
Merus	1	1	6
Merus	2	3	6
Merus	3	2	6
Qilu Pharma	1	2	6
Qilu Pharma	2	4	6
Suzhou Zelgen	1	1	6
Suzhou Zelgen	2	5	6
AP Biosciences	1	2	5
AP Biosciences	2	3	5
EpimAb Biotherap	1	1	5
EpimAb Biotherap	2	4	5
Feng Biosci	1	3	5
Feng Biosci	2	1	5
Feng Biosci	3	1	5
Affimed	1	1	4
Affimed	2	3	4
BeiGene	1	4	4
Genmab, BioNTech	1	1	4
Genmab, BioNTech	2	3	4
Leads Biolabs	2	4	4
Sino Biopharm	1	2	4
Sino Biopharm	2	2	4
Waterstone Hanxbio, HanX Biopharma	1	2	4
Waterstone Hanxbio, HanX Biopharma	2	2	4
Binacea Pharma	1	3	3
Elpiscience	1	2	3
Elpiscience	2	1	3
ImmuneOnco Biopharma	1	2	3
ImmuneOnco Biopharma	2	1	3
Innovent Biologics, Eli Lilly	1	3	3
Mabwell (Shanghai) Biosci	2	3	3
Pfizer	1	3	3
Roche	1	3	3
Takeda	1	1	3
Takeda	2	2	3
University Hospital Tuebingen	1	2	3
University Hospital Tuebingen	2	1	3
ABL Bio	1	2	2
AbbVie	1	2	2
Agenus	1	1	2
Agenus	2	1	2
Astellas	1	2	2
Chong Kun Dang	1	1	2
Chong Kun Dang	2	1	2
Gilead	1	1	2
Gilead	2	1	2
Hanmi, Innovent Biologics	1	1	2
Hanmi, Innovent Biologics	2	1	2
Harbor BioMed, Glenmark	1	1	2
Harbor BioMed, Glenmark	2	1	2
Immunocore	2	1	2
Immunocore	3	1	2
Jiangsu Hengrui Pharma	1	2	2
Kyowa Kirin	1	2	2
L&L Biopharma	1	1	2
L&L Biopharma	2	1	2
Merus, Partner Therap	2	2	2
Phanes Therap	2	2	2
Rongchang Pharma	1	1	2
Rongchang Pharma	2	1	2
Viva Biotech	1	1	2
Viva Biotech	2	1	2
Zhejiang Huahai Pharma	2	2	2
ABL Bio, I‐Mab	1	1	1
ABL Bio, Yuhan Corp	2	1	1
Alligator Biosci, Aptevo Therap	2	1	1
Arbele	1	1	1
Astellas, Amgen	1	1	1
Beijing Mabworks Biotech	1	1	1
Betta Pharma	2	1	1
BioAtla	1	1	1
Biocon	1	1	1
Biocytogen	1	1	1
CSPC Pharma	1	1	1
Celldex	1	1	1
Centessa	2	1	1
Context Therap	1	1	1
Convalife	1	1	1
CytomX, Amgen	1	1	1
D3 Bio	1	1	1
Fosun Pharma	2	1	1
Genmab, AbbVie	2	1	1
German Cancer Research Center, University of Tuebingen	1	1	1
Hanmi	1	1	1
Harbor BioMed	1	1	1
ITabMed	1	1	1
ImmunoGenesis	1	1	1
Incyte	1	1	1
Instill Bio	1	1	1
Keymed Biosciences	2	1	1
LaNova Medicines Limited	2	1	1
Light Chain Biosci	1	1	1
Light Chain Biosci, LamKap Bio	1	1	1
Luye Group	1	1	1
Marengo	2	1	1
Merck (MSD)	2	1	1
Minghui Pharma	2	1	1
NGM Biopharma	2	1	1
OncoC4	2	1	1
Shanghai Junshi Biosci	1	1	1
Sihuan Pharmaceutical	1	1	1
Simcere	1	1	1
Sinocelltech	2	1	1
Sound Biopharma	1	1	1
SparX	1	1	1
Sumgen Biotech	1	1	1
TG ImmunoPharma	1	1	1
Zhejiang Shimai	1	1	1
Zymeworks	1	1	1

**TABLE 9:** Number of trials per status.

Status	Trial count
Recruiting	360
Not yet recruiting	130
Active, not recruiting	77
Completed	68
Terminated	29
Withdrawn	9
Unknown status	4
Enrolling by invitation	2
Suspended	2

## 4 Discussion

The strong focus on immune checkpoint combinations suggests that BsAbs are being explored primarily as enhancers of existing immunotherapy paradigms. While PD‐1/CTLA‐4 and PD‐1/VEGF remain the most frequently investigated targets in recent BsAb clinical trials, other inhibitory pathways within the TME are emerging as equally promising. Dual targeting of PD‐L1 and TGF‐β, a cytokine with potent immunosuppressive and pro‐tumorigenic effects, represents a particularly innovative approach to overcoming resistance to immune checkpoint blockade. The TGF‐β pathway contributes to immune evasion by suppressing cytotoxic T‐cell activation, promoting regulatory T‐cell differentiation, and facilitating tumor fibrosis and angiogenesis [11]. BsAbs such as bintrafusp alfa (M7824), YM101, and BiTP are designed to simultaneously block PD‐L1 signaling and neutralize TGF‐β activity, thereby restoring anti‐tumor immunity. Preclinical studies have shown that these dual inhibitors can convert “cold” tumors into “hot” immune‐inflamed phenotypes, enhancing T‐cell infiltration and promoting durable tumor regression. Clinically, bintrafusp alfa demonstrated early signs of efficacy across multiple solid tumors, including NSCLC and biliary tract cancers. YM101 and BiTP, currently under early‐phase clinical evaluation, have shown encouraging preclinical efficacy and favorable immune activation profiles compared to ICI monotherapies [11, 12, 13, 14, 15]. Meanwhile, the targeted inhibition of oncogenic pathways, particularly *EGFR*, *HER2*, and *ALK*, demonstrates the growing integration of BsAbs into precision oncology approaches.

BsAbs targeting *HER2* and *EGFR*, key oncogenic drivers in multiple tumor types, may benefit from the lessons learned in antibody‐drug conjugates (ADCs) design to improve target selectivity and optimize therapeutic windows [16]. One of the key challenges faced by ADCs is off‐target toxicity, which arises from payload leakage or non‐specific uptake by normal tissues [17]. This challenge is also relevant to BsAbs, particularly in cases where immune activation could lead to excessive inflammation or systemic immune‐related toxicities. The relatively low biomarker‐driven trial designs suggest room for improvement in patient selection. Furthermore, biomarker‐driven strategies can improve the safety profile of BsAbs by ensuring that they only engage with cells that exhibit the target biomarker, reducing the risk of unnecessary immune activation and associated toxicities. Such an approach has already proven successful in ADC development and could be similarly leveraged for BsAbs to enhance efficacy outcomes and streamline regulatory approvals [18, 19, 20].

While BsAb development currently remains concentrated around validated targets such as PD‐1, CTLA‐4, EGFR, and HER2 reflecting both biological rationale and lower regulatory risk—this conservative focus may constrain innovation. Emerging combinations such as PD‐L1 × TGF‐β, PD‐1 × 4‐1BB, and CD47 × PD‐L1 illustrate opportunities to integrate immune modulation with tumor‐intrinsic pathway inhibition [21, 22]. Although challenged by manufacturing and safety considerations, these newer constructs hold promise for overcoming resistance and broadening the therapeutic reach of BsAbs in solid tumors.

Another challenge lies in the complexities of BsAbs manufacturing. Unlike traditional mAbs, BsAbs require precise molecular engineering to ensure stability, correct folding, and balanced binding affinities for both targets [8]. The increased structural complexity adds to production costs and regulatory hurdles. Continued innovation in molecular design can be utilized as a cornerstone for progress. Engineering advancements are crucial to enhance BsAbs' potency while mitigating toxicities. Strategies such as Fc‐engineering, half‐life extension, and conditional activation mechanisms may improve therapeutic performance [8]. Also, the development of combination regimens holds considerable promise. BsAbs may achieve enhanced efficacy when used in combination with ADCs or ICIs. Such combinations could help overcome resistance mechanisms and broaden the therapeutic reach of BsAbs in solid tumors.

Finally, enhanced collaboration between biotech firms and major pharmaceutical companies should be encouraged. Biotechnology companies' emergence as key players in the field indicates a shift toward more specialized and innovative drug development, which should be utilized to accelerate research and commercialization. As the field of BsAbs continues to advance, these innovations will play a critical role in shaping the next generation of immuno‐oncology therapeutics. Effectively addressing these challenges will be key to optimizing BsAbs efficacy and safety, ultimately paving the way for their broader clinical integration.

## 5 Conclusion

In conclusion, our study provides a comprehensive overview of BsAb development in solid tumors, highlighting its rapid expansion since the first approval in 2014. Gastrointestinal cancers remain the most frequently investigated, with ongoing efforts focused on combining BsAbs with immune checkpoint inhibitors and targeting key oncogenic pathways. Continued innovation in target selection, biomarker integration, and bioengineering will be essential to enhance efficacy and safety, shaping the next generation of precision and immune‐oncology therapeutics.

## Author Contributions

**Rafik ElBeblawy:** conceptualization (equal), visualization (equal), writing – original draft (equal), writing – review and editing (equal). **Chinmay Jani:** conceptualization (equal), supervision (equal), writing – review and editing (equal). **Judith Pérez‐Granado:** data curation (equal), methodology (equal), software (equal), validation (equal). **Mark Gramling:** data curation (equal), methodology (equal), software (equal), validation (equal). **Aakash Desai:** conceptualization (equal), project administration (equal), supervision (equal), validation (equal), writing – original draft (equal), writing – review and editing (equal).

## Funding

The authors have nothing to report.

## Conflicts of Interest

The authors declare no conflicts of interest.

## Data Availability

The data that support the findings of this study are available from the corresponding author upon reasonable request.
